# Complete genome sequence of MP2445, a mycobacteriophage that can infect *Mycobacterium bovis* BCG and *Mycobacterium fortuitum*

**DOI:** 10.1128/mra.00080-26

**Published:** 2026-04-20

**Authors:** Xiaoya Zhu, Bo Xu, Jiawen Liu, Xuanran Chen, Furong Ma, Zhengzhong Xu, Xinan Jiao, Xiang Chen, Chengkun Zheng

**Affiliations:** 1 Jiangsu Key Laboratory of Zoonosis, Yangzhou University 38043, Yangzhou, China; 2 Key Laboratory of Prevention and Control of Biological Hazard Factors (Animal Origin) for Agrifood Safety and Quality, the Ministry of Agriculture and Rural Affairs, Yangzhou University 38043, Yangzhou, China; 3 Jiangsu Interdisciplinary Center for Zoonoses and Biosafety, Yangzhou University 38043, Yangzhou, China; Queens College Department of Biology 171429, Queens, New York, USA

**Keywords:** mycobacteriophage, genome, *Mycobacterium*

## Abstract

Mycobacteriophage MP2445 exhibits siphovirus morphology and infects *Mycobacterium smegmatis*, *Mycobacterium bovis* BCG, and *Mycobacterium fortuitum*. Its genome is 50,711 bp in length, has a GC content of 63.34%, and contains 78 protein-coding sequences and one tRNA. MP2445 shares the highest nucleotide identity with phages in the A2 subcluster.

## ANNOUNCEMENT

**Fig 1:**
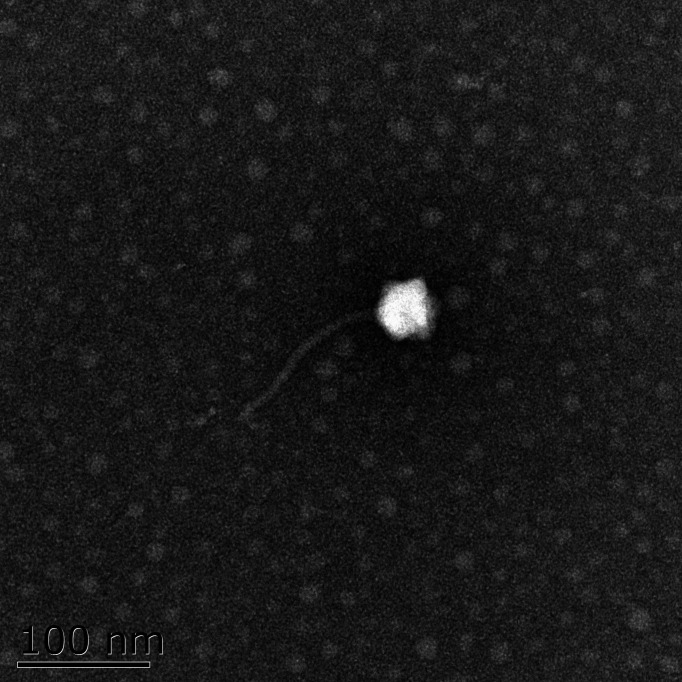
The morphology of mycobacteriophage MP2445 under transmission electron microscopy. Phage particles were negatively stained with 2% phosphotungstic acid and observed using a Tecnai 12 transmission electron microscope at an accelerating voltage of 120 kV. Magnification, ×97,000; scale bar, 100 nm.

Tuberculosis (TB), caused by the *Mycobacterium tuberculosis* complex (MTBC), remains a major global health threat, with an estimated 10.7 million new cases and 1.23 million deaths worldwide in 2024 (1, 2). *Mycobacterium bovis*, a key member of the MTBC, infects humans, cattle, and other mammals, posing a serious zoonotic threat (3). Additionally, *Mycobacterium fortuitum*, a non-tuberculous mycobacterium, can cause refractory infections in the lungs, skin, and other sites, presenting significant risks to human health (4). Phage therapy offers a promising alternative for treating antibiotic-resistant TB and non-tuberculous mycobacterial infections (5). The isolation and characterization of novel phages are crucial for advancing phage-based therapies. Here, we report the genome of mycobacteriophage MP2445, isolated from park soil in Yangzhou, China (coordinates: 119.429036°E, 32.365174°N). This phage infects *Mycobacterium smegmatis*, *M. bovis* BCG, and *M. fortuitum*. Electron microscopy images revealed that MP2445 is a long-tailed phage with an icosahedral head structure (Fig. 1).

MP2445 was isolated using an enrichment culture method. Briefly, 10 g of soil was mixed with 30 mL of Middlebrook 7H9 broth (BD Difco, USA); 2 mL of a stationary-phase culture of *M. smegmatis* mc²155 was then added. The mixture was incubated at 37°C with shaking (160 rpm) for 48 h. After incubation, the culture was centrifuged at 8,000 rpm for 10 min at room temperature, and the supernatant was filtered through a 0.22-μm sterile filter (BIOFIL, Guangzhou, China). The filtrate was then plated using the double-layer agar method on Middlebrook 7H10 agar (BD Difco, USA) inoculated with *M. smegmatis* mc²155. Phage purification was carried out through five rounds of single-plaque isolation to ensure purity. Phage DNA was extracted using the phenol-chloroform method. DNA quality was assessed by 1% agarose gel electrophoresis and a NanoPhotometer spectrophotometer (IMPLEN, CA, USA). DNA concentration was determined using a Qubit 3.0 Fluorometer (Life Technologies, CA, USA). Sequencing libraries were prepared with the TruSeq DNA PCR-Free Library Preparation Kit (Illumina, USA) following the manufacturer’s instructions. Paired-end sequencing (2 × 150 bp) was conducted on an Illumina NovaSeq X Plus platform, generating 3.7 million raw reads. Raw reads were quality-controlled and adapter-trimmed using fastp v0.23.2 (6). The genome was *de novo* assembled with Unicycler v0.5.0 (7). Genome ends were determined using PhageTerm (8). Genome annotation was carried out using Prokka v1.14.6 (9).

The genome of MP2445 is a linear double-stranded DNA molecule of 50,711 bp with a GC content of 63.34%. PhageTerm analysis identified a cos-type packaging mechanism with 10-base 3′ cohesive ends (5′-CGGTCGGTTA-3′), consistent with subcluster A2 mycobacteriophages (10, 11). BLASTn analysis revealed that MP2445 shares the highest nucleotide identity (91% coverage and 90.71% identity) with the mycobacteriophages QueenBeesly (GenBank accession no.: MH697591.1) and FiringLine (GenBank accession no.: MT498060.1), both of which belong to the A2 subcluster. A total of 78 protein-coding genes and one tRNA gene were predicted, covering 88.23% of the genome. No rRNA or tmRNA genes were detected. No antibiotic resistance genes or virulence factors were detected when scanning the CARD (12) and VFDB (13) databases.

## Data Availability

The genome sequence has been deposited in GenBank under the accession no. PX794726.2, and the raw sequences have been deposited in the Sequence Read Archive under the accession no. SRR36709797.
